# Nanometer patterning of water by tetraanionic ferrocyanide stabilized in aqueous nanodrops†
†Electronic supplementary information (ESI) available: Complete citation for ref. 6, 43, 52 and 54, expanded region of nESI spectra in Fig. 1, comparison of individual radial distribution functions and atomic coordinates for the lowest energy Fe(CN)_6_^4–^(H_2_O)_160_ structure along the molecular dynamics trajectory illustrated in Fig. 7. See DOI: 10.1039/c6sc03722d



**DOI:** 10.1039/c6sc03722d

**Published:** 2016-10-17

**Authors:** Matthew J. DiTucci, Evan R. Williams

**Affiliations:** a Department of Chemistry , University of California , B42 Hildebrand Hall , Berkeley , CA 94270 , USA . Email: erw@berkeley.edu

## Abstract

Formation of the small, highly charged tetraanion ferrocyanide, Fe(CN)_6_^4–^, stabilized in aqueous nanodrops is reported. Ion–water interactions inside these nanodrops are probed using blackbody infrared radiative dissociation, infrared photodissociation (IRPD) spectroscopy, and molecular modeling in order to determine how water molecules stabilize this highly charged anion and the extent to which the tetraanion patterns the hydrogen-bonding network of water at long distance. Fe(CN)_6_^4–^(H_2_O)_38_ is the smallest cluster formed directly by nanoelectrospray ionization. Ejection of an electron from this ion to form Fe(CN)_6_^3–^(H_2_O)_38_ occurs with low-energy activation, but loss of a water molecule is favored at higher energy indicating that water molecule loss is entropically favored over loss of an electron. The second solvation shell is almost complete at this cluster size indicating that nearly two solvent shells are required to stabilize this highly charged anion. The extent of solvation necessary to stabilize these clusters with respect to electron loss is substantially lower through ion pairing with either H^+^ or K^+^ (*n* = 17 and 18, respectively). IRPD spectra of Fe(CN)_6_^4–^(H_2_O)_*n*_ show the emergence of a free O–H water molecule stretch between *n* = 142 and 162 indicating that this ion patterns the structure of water molecules within these nanodrops to a distance of at least ∼1.05 nm from the ion. These results provide new insights into how water stabilizes highly charged ions and demonstrate that highly charged anions can have a significant effect on the hydrogen-bonding network of water molecules well beyond the second and even third solvation shells.

## Abstract 2

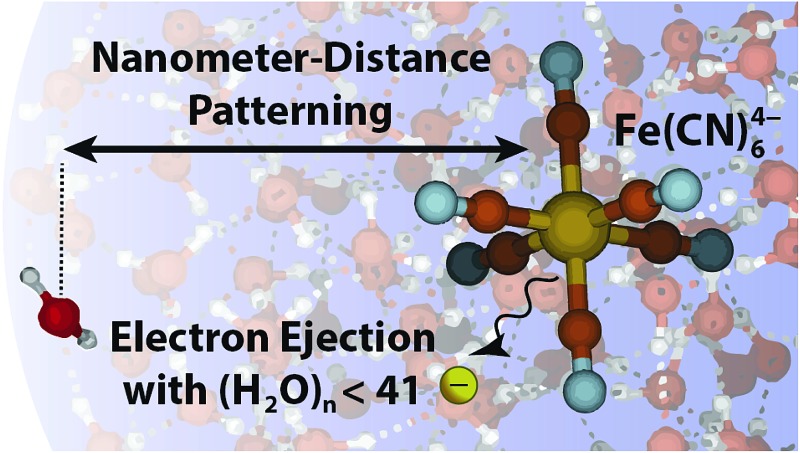
Formation of the small, highly charged tetraanion ferrocyanide, Fe(CN)_6_^4–^, stabilized in aqueous nanodrops and its influence to the surrounding hydrogen-bonding network of water is reported.

## 1 Introduction

Multiply charged anions (MCAs) are ubiquitous in the condensed phases where they play important roles in the chemistry of materials and biology. Without stabilizing interactions with other molecules, such as solvents or counterions, small, highly charged anions can be unstable in isolation owing to strong coulombic repulsion between charges.^1,2^ For example, the sulfate dianion constitutes 7.7% of dissolved inorganics in global seawaters and is found in many natural minerals, such as barite (BaSO_4_), celestine (SrSO_4_) and anglesite (PbSO_4_).^3,4^ It plays an important role in the anaerobic respiration of sulfate-reducing bacteria, such as *Desulfovibrio gigas*, and in the atmospheric chemistry of aerosol radiative forcing, which has an impact on global climate changes.^5,6^ Similarly, the phosphate trianion is stable in basic aqueous solutions and its dianionic conjugate acid acts as a buffer in eukaryotic cells.^7^ However, isolated SO_4_^2–^ and PO_4_^3–^ are metastable and decay by electron ejection with calculated lifetimes of 1.6 × 10^–10^ s and 1.2 × 10^–14^ s, respectively.^8,9^

The stabilities of MCAs within a given charge state generally increase with molecular size due to a greater separation between charges. Excess electrons in MCAs can be bound by a repulsive Coulomb barrier, which manifests as a result of the constructive potentials from short-range electron-nucleus attraction and long-range electron–electron repulsion.^10–12^ For dicarboxylate dianions, ^–^O_2_C(CH_2_)_*x*_CO_2_^–^, the electron affinity lessens with decreasing *x* due to an increase in the repulsive electron–electron potential.^13^ The electron affinity is negative for *x* ≤ 2 (∼5.2 Å). As a result, many large MCAs, such as fullerenes,^14,15^ peptides,^16^ proteins,^17^ and DNA^18,19^ are stable as isolated ions, whereas many small MCAs are not.

Small MCAs can be stabilized by interactions with solvent molecules.^20^ High-valency anions in solvated clusters can be generated *via* electrospray ionization,^20–22^ which provides a useful method for studying the role of solvation in the stabilities of MCAs as well as the distal extent of ion–water interactions. Wang and co-workers used photoelectron spectroscopy to investigate hydrated sulfate, SO_4_^2–^(H_2_O)_*n*_, and oxalate, C_2_O_4_^2–^(H_2_O)_*n*_, clusters, which both require solvation by at least three water molecules to be thermodynamically stable.^23,24^ Extrapolation of experimental data suggests SO_4_^2–^(H_2_O)_*n*_ with *n* = 1, 2 are unstable by –0.9 and –0.2 eV, respectively.^20^ However, formation of SO_4_^2–^(H_2_O)_2_ *via* collisionally-induced dissociation from *n* = 4 has been reported by Blades and Kebarle suggesting the cluster has a sufficient lifetime to be observed using mass spectrometry.^25^

The primary dissociative pathway for ion-containing clusters depends on the identity of the solvated ion, the cluster size and the internal energy of the cluster. For example, blackbody infrared radiative dissociation (BIRD) of SO_4_^2–^(H_2_O)_*n*_ at 21 °C with *n* ≤ 5 predominately results in charge separation to form OH^–^(H_2_O)_*k*_ and HSO_4_^–^(H_2_O)_*n*–*k*–1_ product ions.^26^ However, loss of a water molecule is entropically favored, and as a result, SO_4_^2–^(H_2_O)_*n*_ clusters with *n* < 5 become dominant products under higher energy activation conditions. For this reason, a critical cluster size, *n*_c_, has been defined as the size at which charge separation becomes energetically favored over the loss of a water molecule.^27^ Recent results show hydrates of the ferricyanide trianion, Fe(CN)_6_^3–^(H_2_O)_*n*_, are unstable to electron ejection with a critical cluster size of *n*_c_ = 8, which IRPD spectroscopy and theory show is still within the first solvation shell of the ion.^21^ Although isolated P_3_O_9_^3–^ and Co(NO_2_)_6_^3–^ have been reported previously,^28^ subsequent experiments show *n*_c_ = 6 is required to stabilize P_3_O_9_^3–^ with respect to charge separation and no evidence for isolated Co(NO_2_)_6_^3–^ was found under identical experimental conditions for forming P_3_O_9_^3–^(H_2_O)_*n*_ and Fe(CN)_6_^3–^(H_2_O)_*n*_.^21^ Therefore, Fe(CN)_6_^3–^ and P_3_O_9_^3–^ are the only small, high-charge density trianions that have been observed in gas phase aqueous clusters and at minimum require first-shell hydration for thermodynamic stability.

The distance to which ion-induced solvent orientation can extend has been studied in solution using multiple techniques, including diffraction,^29,30^ spectroscopy^31–33^ and theory.^34,35^ Although ion-induced structuring of water molecules within the coordination shell is unambiguous, there have been conflicting interpretations amongst these results as to whether ion–water interactions can extend beyond the first solvation shell. For example, recent dielectric relaxation studies on hydrated phosphates indicate interactions extend into a full second solvation shell.^33^ Although previous neutron diffraction studies have revealed extended ion–water interactions,^36^ experiments with hydrated phosphate salts show no evidence for ionic influences beyond direct coordination to the ion.^30^ The dynamic nature of water molecules makes probing weak ion-induced structural effects beyond a rigid inner shell a challenging endeavor. Measurements of MCAs in aqueous solution contain contributions from several counterions, which complicates the ability to assign interactions between water molecules and a single ion. Furthermore, many condensed phase techniques rapidly lose sensitivity with decreasing concentration such that uncoordinated water molecules are scarce and long-distance orientation effects from a single ion cannot be inferred.

The structure of size-selected (H_2_O)_*n*_ aqueous clusters in the gas phase can be studied using vibrational spectroscopy in the O–H stretching region. Water molecules at the surface of neutral clusters are free to orient with non-bonded hydrogens facing away from the cluster, which has been observed experimentally by the unique absorption band at ∼3700 cm^–1^.^37,38^ Using IRPD spectroscopy of mass-selected, ion-containing water clusters, orientation effects on water by a single solvated ion free of any counterion contributions can be measured.^21,39,40^ The sensitivity of this technique is not limited by the ratio of water molecules to ions. As a result, ion–water interactions can be measured to a hypothetical infinite dilution. IRPD spectra for SO_4_^2–^ and Fe(CN)_6_^3–^ show that the non-bonded O–H stretch is absent with droplet radii up to ∼0.7 and 0.8 nm, respectively, as a result of long-distance orientation effects by the MCAs.^21,39^

The tetraanion ferrocyanide, Fe(CN)_6_^4–^, is stable in aqueous solution, where it is commonly used as a probe for monitoring intracellular redox events in living cells.^41^ It is also a precursor to the pigment Prussian blue, Fe_4_[Fe(CN)_6_]_3_, which is used also as a medical treatment for internal radioactive Cs^+^ and Tl^+^ poisoning and is being considered for clean-up of chemically-polluted natural waters caused by nuclear contamination.^42–44^ Experiments on this ion in water have revealed a distinct inner solvation shell around ferrocyanide, but no information has been reported about how this anion influences the structure of water beyond the first solvation shell.^45–48^ The direct measurement of extended ion–water interactions by an individual tetraanionic species is not feasible in the condensed phase due to the low concentrations necessary for hydration shells to exist unperturbed by counterions. However, the stability afforded by aqueous gas phase clusters and the sensitivity of IRPD spectroscopy to the orientation of surface water molecules provide a well-suited method for studying this high charge density tetraanion.

Here, the dissociation pathways and ion–solvent interactions in hydrated clusters of Fe(CN)_6_^4–^ are investigated using BIRD, IRPD spectroscopy and molecular modeling. Remarkably, Fe(CN)_6_^4–^(H_2_O)_*n*_ with *n* = 41 is required for thermodynamic stability, although metastable clusters as small as *n* = 37, which dissociate *via* electron ejection, are observed. These findings illustrate that stability to electron ejection is achieved only through solvation with a nearly complete second hydration shell and therefore provides a direct observation of long-distance interactions between the ion and solvent. The spectroscopic signatures in the O–H oscillator region show ion-induced patterning of the hydrogen-bonding network can extend beyond a nanometer distance from the ion, corresponding to the fourth solvation shell. In combination with ion–solvent interactions in lower valency anions, a minimum Coulomb potential required for orienting surface water molecules in clusters can be determined. This is the first report of a critical cluster size for a small hydrated tetraanion and is the smallest, highest charge density tetraanion that has been formed in the gas phase.

## 2 Experimental and computational methods

All experimental data were obtained using a home-built 7.0 T Fourier transform ion cyclotron resonance (FT-ICR) mass spectrometer based on an instrument described elsewhere,^49^ which has been upgraded to incorporate a 7 T magnet. Hydrated ions are formed by nanoelectrospray ionization (nESI) of 10 mM aqueous solutions of potassium hexacyanoferrate(ii) trihydrate (Sigma Aldrich, St. Louis, MO) with purified water from a Milli-Q system (Millipore, Billerica, MA). Solutions are loaded into borosilicate capillaries with tips that have been pulled to an inner diameter of ∼1 μm. A platinum wire in contact with the solution is held at a constant potential of ∼700 V with respect to a heated metal capillary at the entrance of the mass spectrometer. Ions are guided *via* electrostatic lenses through five stages of differential pumping into the ion cell, which is enclosed by a copper jacket that is temperature-controlled to 133 K by a flow of liquid nitrogen and is thermalized for at least 8 h prior to data acquisition.^50^ A pulse of dry nitrogen gas is introduced to bring the pressure of the vacuum chamber containing the ion cell to ∼2 × 10^–6^ Torr, which helps to both trap and thermalize the ions. After an ∼8 s pump down period following the pulse gas, the pressure in the chamber decreases to ∼2 × 10^–9^ Torr. Precursor ions of interest are subsequently isolated using a notched stored waveform inverse Fourier transform excitation.

IRPD spectra between 2800 and 3800 cm^–1^ are measured with infrared photons from a tunable OPO/OPA system (LaserVision, Bellevue, WA) pumped by the 1064 nm fundamental of a Nd:YAG laser (Continuum Surelight I-10, Santa Clara, CA) operating at a 10 Hz repetition rate. Mass-selected precursor ions isolated in the ion cell are irradiated for between ∼0.25–1.0 s in order to produce substantial, but not complete, dissociation of the isolated aqueous cluster. A first-order rate constant is obtained from the relative abundances of precursor and product ions after photodissociation. The IRPD rate constants from laser irradiation are corrected for frequency dependent variations in laser power as well as dissociation due to BIRD.^51^

Molecular dynamics trajectories were obtained using Desmond 3.1 (Schrödinger, Inc., Portland, OR). The isolated ferrocyanide complex was first optimized using Q-Chem 4.0 (ref. 52) (Q-Chem, Inc., Pittsburgh, PA) at the B3LYP/LACVP++** level of theory. This optimization uses a confined electronic structure that does not permit electron ejection. Water molecules were added thereafter with the optimized ferrocyanide complex in the center to model Fe(CN)_6_^4–^(H_2_O)_160_. During the simulation, the Fe–C force constants were increased in order to avoid cyano ligand loss, which does not occur experimentally for nanodrops of this size, but has been observed previously in QM/MM simulations.^48^ The ion was not confined to the center of the aqueous cluster during the dynamics trajectories. An initial geometry relaxation was performed followed by equilibration with a 1 ns stochastic dynamics trajectory at 133 K using the OPLS 2005 force field with 1.0 fs time steps. In order to increase the conformational space explored in these trajectories, a series of annealing cycles were used to generate low-energy conformers. The upper temperature was chosen such that evaporation does not occur and water molecules can readily exchange between hydration shells. Each cycle consists of 200 ps at 195 K followed by gradual linear cooling over 300 ps to 135 K. The system is then equilibrated at 135 K for 1 ns followed by an additional 1 ns trajectory, from which five structures are taken in regular intervals. A comparison of the radial distribution functions for individual structures along the simulation shows that the hydrogen-bonding network of Fe(CN)_6_^4–^(H_2_O)_160_ is not static within the final 1 ns of an annealing cycle at 133 K (Fig. S1†). This procedure is repeated 20 times in order to generate 100 low-energy structures for Fe(CN)_6_^4–^(H_2_O)_160_. The atomic coordinates from these structures are used to measure radial distribution functions, dipole orientations and estimate the ion-surface distance for Fe(CN)_6_^4–^(H_2_O)_160_. The same procedure for simulated annealing molecular dynamics was also used to generate 100 low-energy structures for (H_2_O)_165_, which are used to measure water molecule dipole orientations in neutral clusters.

## 3 Results and discussion

### 3.1 Hydrated ion formation

**Fig. 1:**
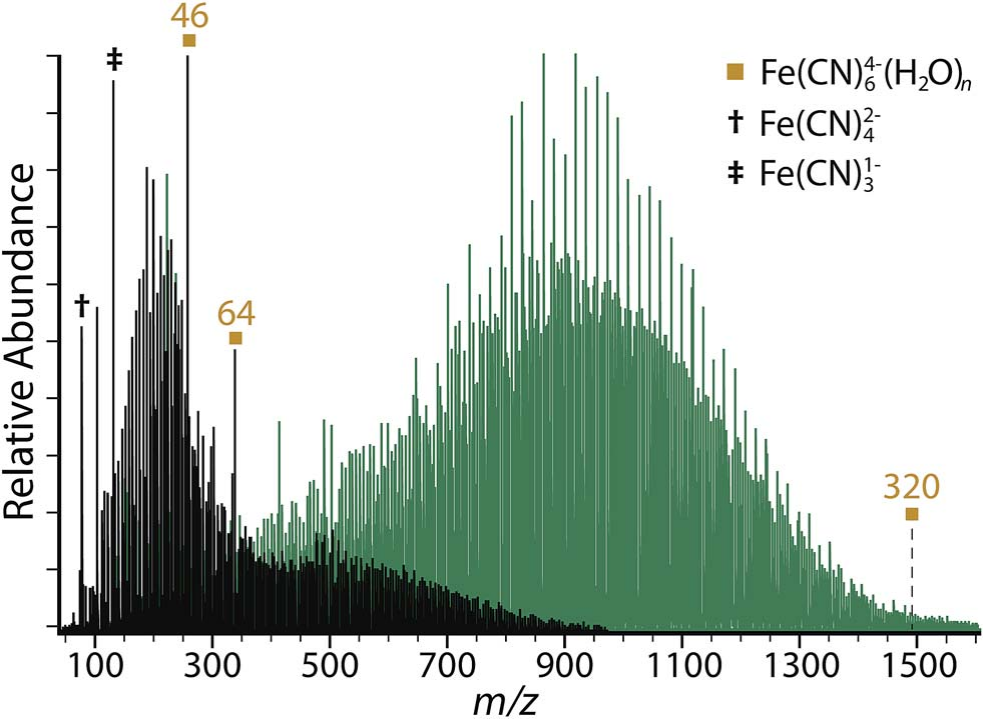
Nanoelectrospray ionization mass spectra obtained from 10 mM aqueous solutions of K_4_Fe(CN)_6_ with instrumental conditions tuned for small (black) and large clusters (green). Magic number clusters for Fe(CN)_6_^4–^(H_2_O)_*n*_ are observed at *n* = 46 and 64. Bare species with no hydrates include Fe(CN)_4_^2–^ (†) and Fe(CN)_3_^–^ (‡).

**Fig. 2:**
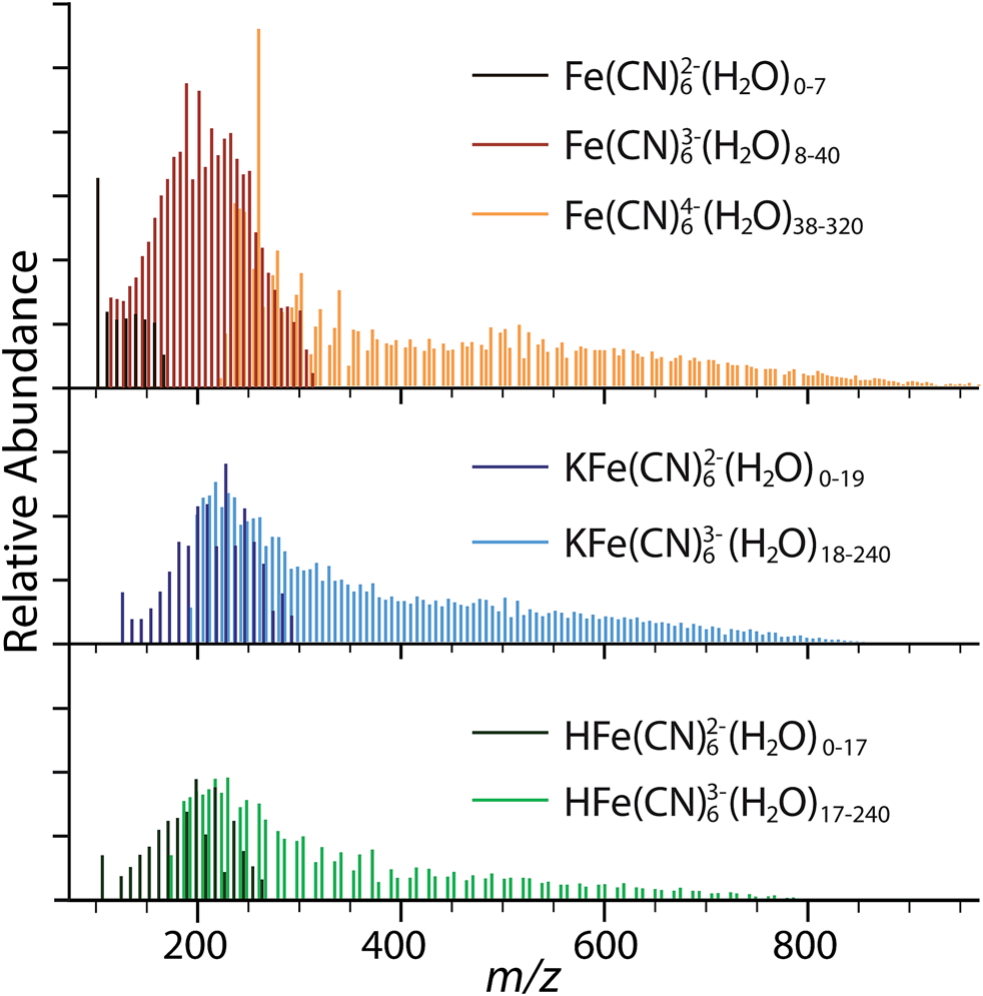
Hydrated ion distributions in nanoelectrospray ionization mass spectrum obtained from 10 mM aqueous solution of K_4_Fe(CN)_6_ with instrumental conditions tuned for small clusters. The most abundant ions are obtained from the mass spectral data and are plotted separately. Peaks from overlapping clusters are not included in these isolated spectra and account for gaps in the distributions for Fe(CN)_6_^4–^ and HFe(CN)_6_^3–^.

Several different hydrated ions are observed in the nESI mass spectra of 10 mM aqueous solutions of K_4_Fe(CN)_6_ (Fig. 1), including the tetraanion ferrocyanide, Fe(CN)_6_^4–^(H_2_O)_*n*_. The smallest ferrocyanide cluster produced under a range of instrumental conditions is *n* = 38, but clusters with *n* > 320 can be readily formed by reducing voltages to source optics, decreasing the temperature of the entrance capillary, and by optimizing the excitation waveform to enhance detection of larger clusters. Expanded regions of the mass spectra tuned for both small and large cluster sizes show the onset of ferrocyanide clusters at *n* = 38 and the presence of protonated and potassiated adducts (Fig. S2†). Three isolated species without hydrate distributions are observed, Fe(CN)_5_^2–^ (*m*/*z* = 92.9), Fe(CN)_4_^2–^ (*m*/*z* = 79.9) and Fe(CN)_3_^–^ (*m*/*z* = 133.9), which are formed *via* sequential ligand loss from the ferricyanide dianion.^21^ A summary of all hydrated species formed by nESI is given in Table 1 and the isolated distributions of each hydrated ion are shown in Fig. 2.

**Table 1:** Hydrated species formed by nESI of 10 mM K_4_Fe(CN)_6_

Species formula	Smallest *n*	Largest *n*
Fe(CN)_6_^4–^(H_2_O)_*n*_	38	>320
Fe(CN)_6_^3–^(H_2_O)_*n*_	8	40
Fe(CN)_6_^2–^(H_2_O)_*n*_	0	7
H_2_Fe(CN)_6_^2–^(H_2_O)_*n*_	0	>150
HFe(CN)_6_^3–^(H_2_O)_*n*_	17	>240
HFe(CN)_6_^2–^(H_2_O)_*n*_	0	17
K_2_Fe(CN)_6_^2–^(H_2_O)_*n*_	0	>150
KFe(CN)_6_^3–^(H_2_O)_*n*_	18	>240
KFe(CN)_6_^2–^(H_2_O)_*n*_	0	19

The smallest Fe(CN)_6_^4–^(H_2_O)_*n*_ cluster formed by nESI is *n* = 38, whereas the largest Fe(CN)_6_^3–^(H_2_O)_*n*_ is *n* = 40. Similarly, the smallest Fe(CN)_6_^3–^(H_2_O)_*n*_ cluster is *n* = 8 and the largest Fe(CN)_6_^2–^(H_2_O)_*n*_ is *n* = 7. This is consistent with the previous results for Fe(CN)_6_^3–^(H_2_O)_*n*_, which show that electron ejection occurs for *n* ≤ 8.^21^ The absence of dianionic Fe(CN)_6_^2–^(H_2_O)_*n*_ clusters with *n* > 8 indicates this species is not present in solution, but is instead formed *via* gas phase dissociation of Fe(CN)_6_^3–^(H_2_O)_*n*_ with *n* ≥ 8. Similarly, the Fe(CN)_6_^3–^(H_2_O)_*n*_ distribution from *n* = 8–40 likely originates *via* electron ejection from larger unstable tetraanion ferrocyanide clusters. There is a similar correlation between charge states for the adducted species. Based on the distributions, electron ejection likely occurs from HFe(CN)_6_^3–^(H_2_O)_*n*_ near *n* ≃ 17 and for KFe(CN)_6_^3–^(H_2_O)_*n*_ with *n* ≃ 19. The cluster size distributions indicate that the lower charge state species are formed in evaporating electrospray droplets through dissociative pathways and are not abundant in aqueous solutions of K_4_Fe(CN)_6_, which primarily contain the ferrocyanide tetraanion and trianionic ion pairs formed by protonation and potassiation of Fe(CN)_6_^4–^.

The smallest cluster sizes formed by nESI are smaller for HFe(CN)_6_^3–^ (*n* = 17) and KFe(CN)_6_^3–^ (*n* = 18) ion-paired species than Fe(CN)_6_^4–^. The greater stability associated with proton adduction compared to potassium ion adduction is likely due to more favorable interactions with the higher charge density of H^+^ with respect to K^+^. These clusters are larger than the smallest clusters of Fe(CN)_6_^3–^ (*n* = 8), which indicates that the ferricyanide trianion is intrinsically more stable than the trianions resulting from cation adduction to Fe(CN)_6_^4–^. Similarly, we see electron ejection from HFe(CN)_6_^3–^ and KFe(CN)_6_^3–^ results in dianionic species that are stable as bare ions as a result of the stability gained through ion-pairing. Therefore, the stability gained by cation adduction decreases the onset of dissociation and the critical cluster size for MCAs.

There are two magic number clusters for ferrocyanide at *n* = 46 and 64. Although peak overlap occurs for Fe(CN)_6_^4–^ with HFe(CN)_6_^3–^ and H_2_Fe(CN)_6_^2–^ (Fig. S2†), which are isobaric to within 0.01 and 0.03 *m*/*z* for specific cluster sizes, these magic number clusters are confirmed to originate from the tetraanion based on the isotope spacing. Although the structural origin of the magic number cluster stabilities is unknown, infrared photodissociation studies have suggested that complex clathrate structures may form for clusters of this size, which optimize hydrogen bonding and ultimately lead to enhanced stability.^21,53,54^

### 3.2 Critical cluster size and dissociation pathways

**Fig. 3:**
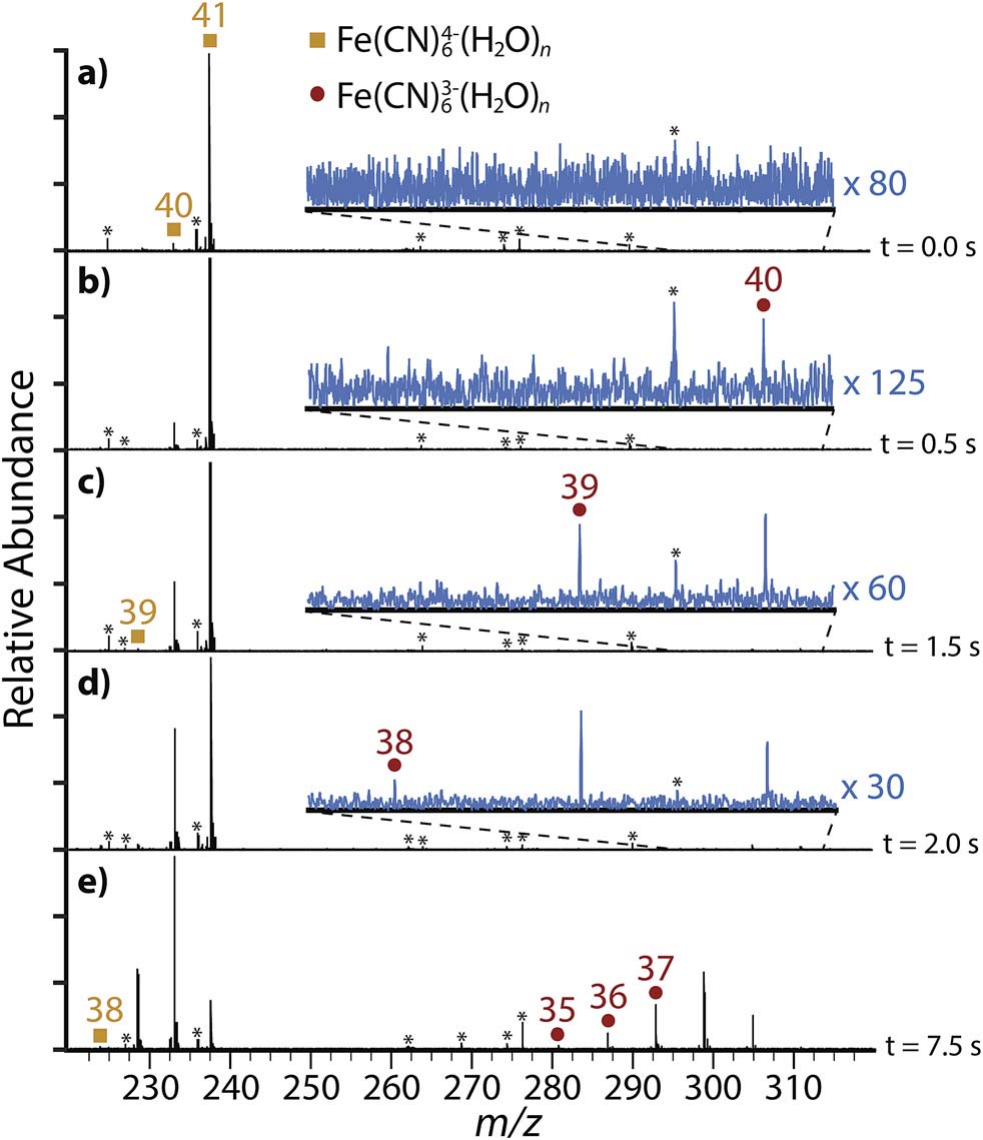
Blackbody infrared dissociation spectra of (a) Fe(CN)_6_^4–^(H_2_O)_41_ showing product ions after (b) 0.5 s, (c) 1.5 s, (d) 2.0 s, (e) 7.5 s. Frequency noise is labeled with an asterisk (*).

The smallest Fe(CN)_6_^4–^(H_2_O)_*n*_ cluster that is formed directly by nESI using a variety of source conditions is *n* = 38. In order to determine the dissociation pathways for this cluster, the isolated precursor Fe(CN)_6_^4–^(H_2_O)_41_ (Fig. 3a) was dissociated using BIRD at 133 K (Fig. 3b–e). This cluster size was chosen in order to both maximize signal abundance and to avoid signal overlap caused by the clusters containing HFe(CN)_6_^3–^ and H_2_Fe(CN)_6_^2–^ (Fig. S2†). Dissociation from Fe(CN)_6_^4–^(H_2_O)_41_ occurs exclusively through loss of a single water molecule (reaction (1)):

1 Fe(CN)_6_^4–^(H_2_O)_*n*_ → Fe(CN)_6_^4–^(H_2_O)_*n*–1_ + H_2_O

After 0.5 seconds (Fig. 3b), a low abundance peak corresponding to Fe(CN)_6_^3–^(H_2_O)_40_ is observed. This product cannot come directly from Fe(CN)_6_^4–^(H_2_O)_41_ by direct loss of H_2_O^–^ because the electron affinity of a single water molecule is negative. Therefore, this product is formed *via* electron ejection from Fe(CN)_6_^4–^(H_2_O)_40_ (reaction (2)):

2 Fe(CN)_6_^4–^(H_2_O)_*n*_ → Fe(CN)_6_^3–^(H_2_O)_*n*_ + e^–^

After 1.5 seconds (Fig. 3c), Fe(CN)_6_^3–^(H_2_O)_39_, which can be formed either through loss of a water molecule from Fe(CN)_6_^3–^(H_2_O)_40_ or electron ejection from Fe(CN)_6_^4–^(H_2_O)_39_, starts to appear. Fe(CN)_6_^4–^(H_2_O)_40_ also dissociates *via* water loss to form Fe(CN)_6_^4–^(H_2_O)_39_. Thus, Fe(CN)_6_^4–^(H_2_O)_40_ dissociates *via* two pathways – either through loss of a single water molecule (reaction (1)) or electron ejection (reaction (2)).

The abundance of Fe(CN)_6_^3–^(H_2_O)_40_ is less than that of Fe(CN)_6_^3–^(H_2_O)_39_ after 2.0 s (Fig. 3d). This inversion occurs as a result of only a single precursor dissociating to the former whereas the latter is produced by two dissociative pathways. Fe(CN)_6_^3–^(H_2_O)_38_ begins to appear at 2.0 s and it can also be formed by two precursors. The smallest tetraanion observed from BIRD, Fe(CN)_6_^4–^(H_2_O)_38_, appears after 7.5 s (Fig. 3e) and it exclusively dissociates *via* electron ejection. Therefore, the trianion clusters Fe(CN)_6_^3–^(H_2_O)_*n*_ with *n* ≤ 37 are only formed by sequential water loss from the electron ejection products under low energy ambient blackbody radiation conditions.

The droplet radius for Fe(CN)_6_^4–^(H_2_O)_41_ is ∼0.67 nm, which suggests that valence electrons must delocalize beyond the inner solvation shell for thermodynamic stability. Added stability from a subsequent shell can be due to either the added dielectric shielding by the solvent or an extended delocalization of the electron density into the second solvation shell. Evidence for the latter has been reported for the microsolvated fluoride anion, F^–^(H_2_O)_6_.^55^ Calculations by Canuto *et al.* indicate that the six valence electrons of fluoride are distributed over the entire cluster. Dyson orbitals corresponding to F^–^(H_2_O)_6_ vertical electron detachment energies (VEDE) suggest that the majority of the valence orbital density is delocalized over the water molecules with a maximum of 45% localized on fluorine, which occurs only for a high-energy VEDE.^55^ Therefore, it is possible that delocalization of the orbital density for valence electrons in Fe(CN)_6_^4–^(H_2_O)_*n*_ is required to extend beyond the coordinating hydration shell before the system is stable to electron ejection, which can explain the necessity of *n* ≥ 41 water molecules required in order to fully stabilize Fe(CN)_6_^4–^.

**Fig. 4:**
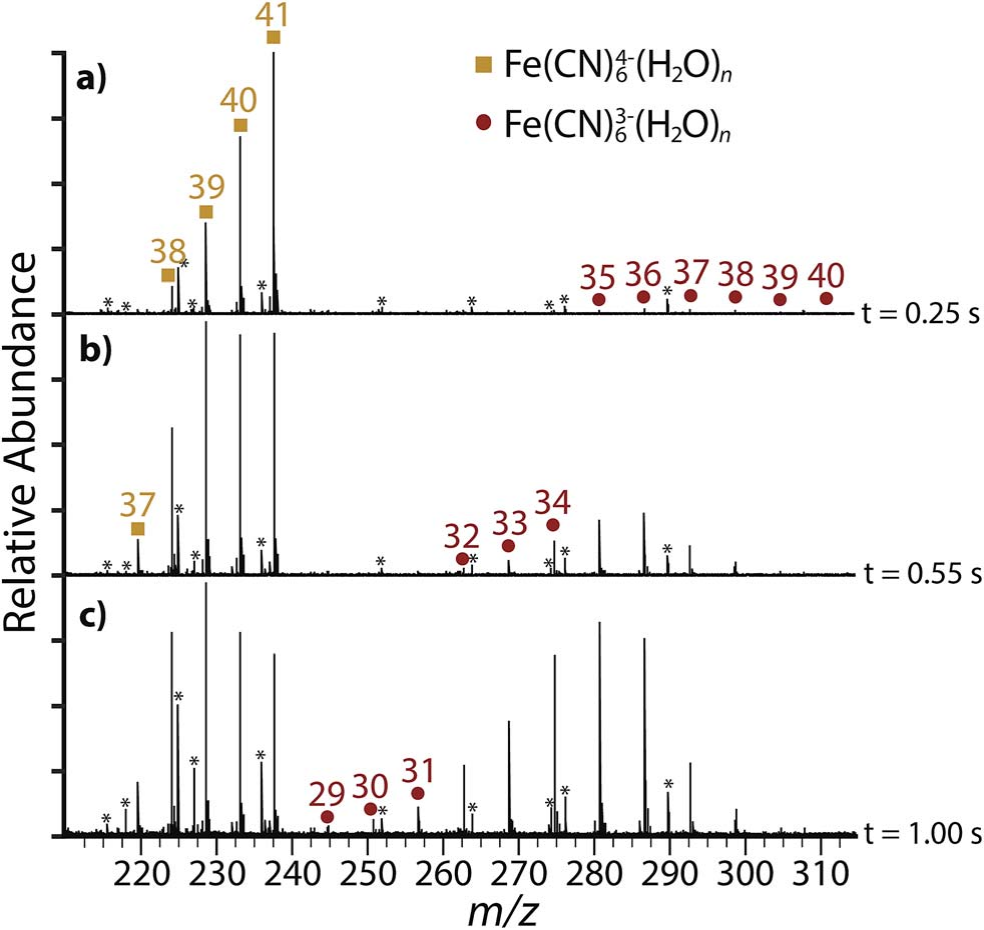
Infrared photodissociation spectra of Fe(CN)_6_^4–^(H_2_O)_41_ at 3521 cm^–1^ showing product ions after (a) 0.25 s, (b) 0.55 s, (c) 1.00 s. Frequency noise is labeled with an asterisk (*).

Although Fe(CN)_6_^3–^(H_2_O)_8_ exclusively dissociates *via* electron ejection under low energy BIRD conditions, water loss to form the *n* = 7 cluster is entropically favored and becomes the major product under higher energy excitation conditions.^21^ To investigate whether the same is true for Fe(CN)_6_^4–^(H_2_O)_*n*_, infrared photons from a tunable laser at 3521 cm^–1^, corresponding to the hydrogen-bonded region, were used to dissociate Fe(CN)_6_^4–^(H_2_O)_41_ for various times (Fig. 4). Sequential water molecule loss to form Fe(CN)_6_^4–^(H_2_O)_38_, the smallest tetraanion observed using BIRD, is visible after only 0.25 s (Fig. 4a). Electron ejection products, Fe(CN)_6_^3–^(H_2_O)_*n*_, are also observed for *n* = 35–40. There is no evidence for electron ejection from Fe(CN)_6_^4–^(H_2_O)_41_, which continues to dissociate exclusively through water loss with higher energy dissociation. After 0.55 s (Fig. 4b) water loss from Fe(CN)_6_^4–^(H_2_O)_38_ occurs to form Fe(CN)_6_^4–^(H_2_O)_37_, which is the smallest tetraanion observed in these experiments. Longer dissociation times up to 1.00 s (Fig. 4c) result only in further sequential water loss from Fe(CN)_6_^3–^(H_2_O)_*n*_. Therefore, water loss is entropically-favored for Fe(CN)_6_^4–^(H_2_O)_38_ and it occurs only under higher energy excitation conditions.

### 3.3 Patterning of water at long distances by Fe(CN)_6_^4–^

**Fig. 5:**
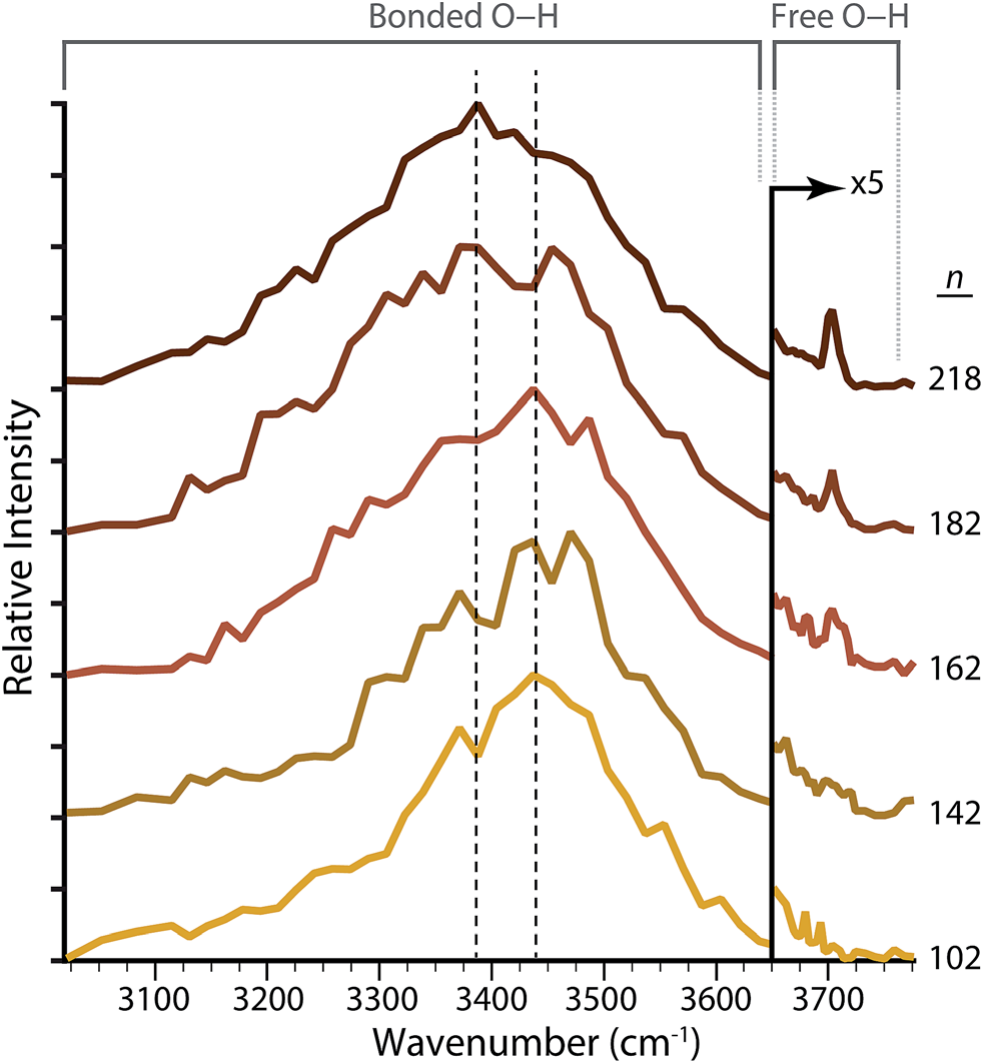
Infrared photodissociation spectra of Fe(CN)_6_^4–^(H_2_O)_*n*_ with *n* between 102 and 218. The spectra have been expanded by 5× above 3650 cm^–1^ in order to more clearly show the free O–H region, where acceptor–acceptor–donor (AAD) water molecules begin to appear for *n* = 162 at 3704 cm^–1^.

To investigate the effect Fe(CN)_6_^4–^ has on the structure of the hydrogen-bonding network of water molecules located remotely from the ion, IRPD spectra of Fe(CN)_6_^4–^(H_2_O)_*n*_ at select sizes between *n* = 102–218 were measured (Fig. 5). There is a broad feature in the spectral region between ∼3000–3600 cm^–1^ that is observed for all cluster sizes. Intensity within this frequency range corresponds to water molecules that donate two hydrogen bonds either to the solvated ion or to adjacent water molecules. These oscillators include three- or four-coordinate water molecules in the bulk of the cluster that accept one (ADD, or acceptor–donor–donor) or two (AADD, or acceptor–acceptor–donor–donor) hydrogen bonds, or two-coordinate water molecules at the surface of the cluster that do not accept any hydrogen bonds (DD, or donor–donor). The maximum of this feature in the bonded O–H region for Fe(CN)_6_^4–^(H_2_O)_*n*_ red shifts from 3438 cm^–1^ (*n* = 102) to 3389 cm^–1^ (*n* = 218), which is indicative of more optimal, stronger hydrogen bonding between water molecules in the larger clusters.

For hydrated clusters with *n* = 102 and 142, intensity in the region between 3600–3800 cm^–1^ is fully attributable to the tail of the fully hydrogen-bonded feature. A band near ∼3700 cm^–1^ is due to water molecules that donate only one hydrogen bond, leaving the second O–H oscillator, commonly referred to as a “free O–H”, at the surface facing outward and away from the cluster. Absence of unique spectral features near ∼3700 cm^–1^ indicates that all water molecules on the surface of the cluster are donating two hydrogen bonds. The spectra for clusters with *n* ≥ 162 have a feature centered at ∼3704 cm^–1^ characteristic of three-coordinate AAD (acceptor–acceptor–donor) water molecules.^53,54,56–60^ The frequency of these oscillators depends on the charge state of the hydrated ion, the extent of hydration and to a lesser extent, the size of the ion.^61,62^ The intensity of this band increases for *n* = 182 and 218, indicating that the surface of these clusters is populated with an increasing number of AAD water molecules.

For cations, the AAD oscillators are observable at the onset of hydration and increase in intensity for larger clusters, whereas for higher valency anions, this stretch is absent until there are a sufficient number of water molecules to effectively shield the ionic potential from water molecules at the cluster surface. The effect of the ion on the hydrogen-bonding network of water molecules in these clusters depends both on the orientation of water molecules directly coordinated to the ion and the electrostatic interaction of the solvated ion with the water dipole, which orients inwards toward the ion for anionic hydrated clusters. As a result, the initial presence of this free O–H stretch for an anionic cluster size indicates the extent of water patterning by the solvated ion. Although a free O–H stretch may appear for select microsolvated cluster sizes as a consequence of the available bonding motifs, *i.e.*, water molecules in a small cluster with no hydrogen-bonding partner accessible, there are no consistent free O–H stretches in the IRPD spectra of (H_2_O)_*n*_^–^ until *n* ≥ 15,^63^ for SO_4_^2–^(H_2_O)_*n*_ until *n* > 43,^39^ and for Fe(CN)_6_^3–^(H_2_O)_*n*_ until *n* ≥ 70.^21^ While the exact location of the electron in (H_2_O)_*n*_ has not yet been resolved, it is evident that all water molecule hydrogen atoms interact with the diffuse electron cloud until *n* ≥ 15.^63^ In contrast, the higher charge state anions are expected to be located closer to the center of the cluster to maximize the stabilizing effects of solvation.

**Fig. 6:**
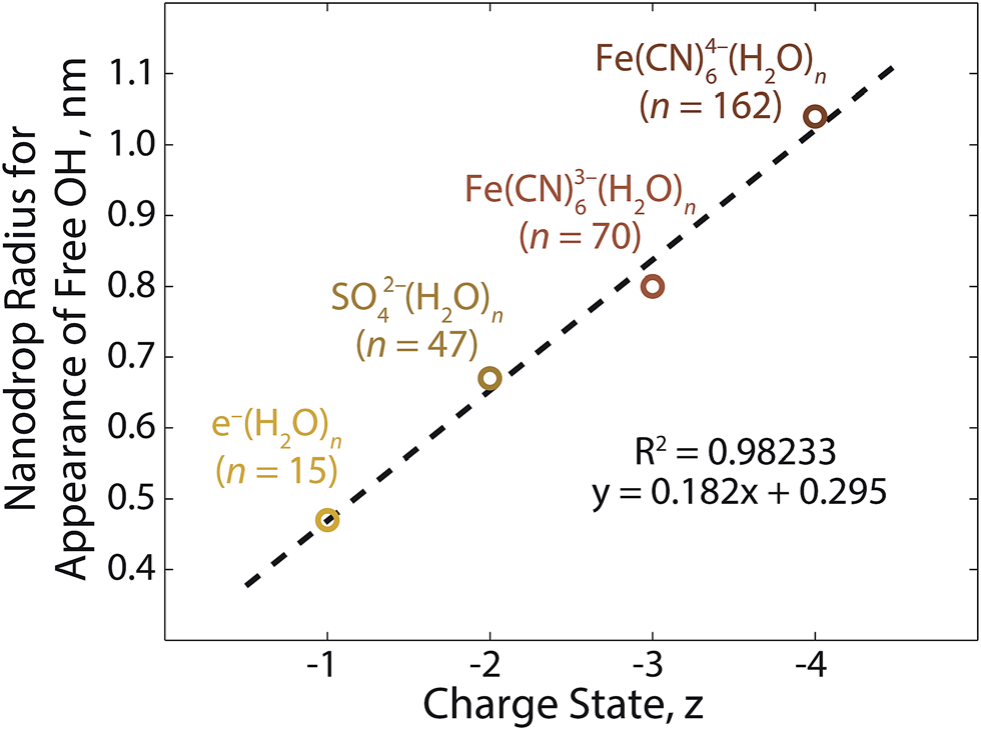
Linear relationship between the nanodrop radius for the onset of acceptor–acceptor–donor (AAD) water molecules at the cluster surface and corresponding anionic charge state.^21,39,63^ The cluster radius is obtained from the water density assuming the nanodrops are spherical. Void space due to the ion is neglected.

In order to ascertain the relationship between ionic charge state and patterning distance, the radii of clusters for which AAD water molecules begin to appear as a function of the anionic charge state is shown in Fig. 6. The four anionic charge states follow a linear trend with distance. This is the behavior expected from a Coulombic potential, *V*_Coulomb_, produced by a point charge, *Q* (eqn (3)):3
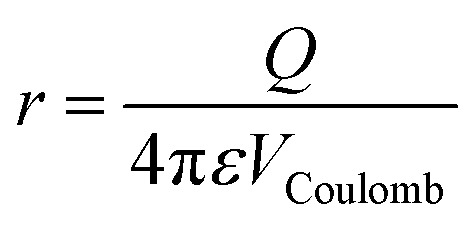
where *r* is the radius of the hydrated cluster and *ε* is the permittivity of water. Because *r* is proportional to *Q*, the potential, *V*_Coulomb_, is expected to be roughly constant across the four anionic charge states for the cluster size where AAD water molecules appear. Using the permittivity of ice extrapolated to 133 K, this potential is calculated to be (2.4 ± 0.6) × 10^–13^ J C^–1^. This is the minimum potential required in order to orient the water molecules inward and prevent the emergence of free O–H oscillators at the cluster surface.

**Fig. 7:**
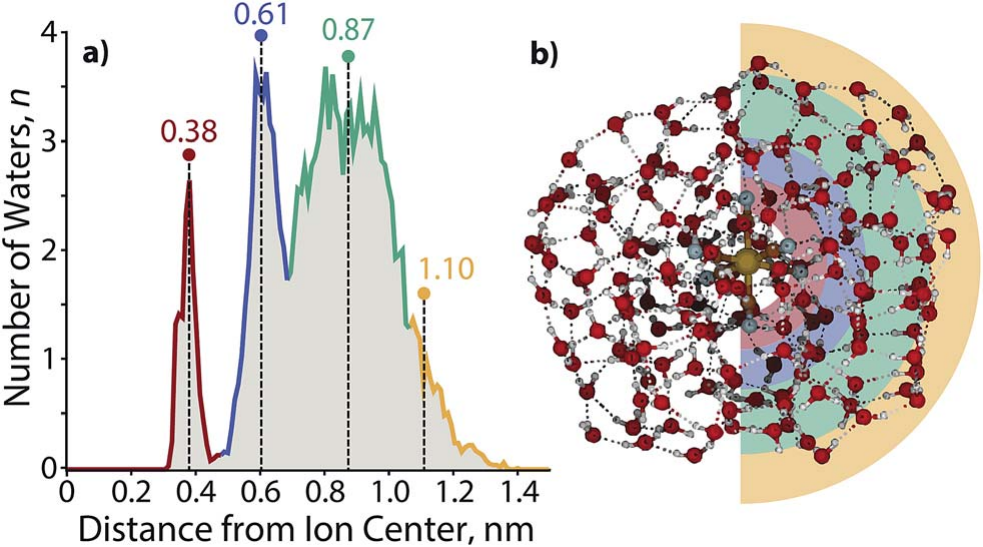
(a) Radial distribution plot illustrating the structure for solvation shells around Fe(CN)_6_^4–^(H_2_O)_160_. Water molecules are counted within a bin width for a radial thickness of 0.01 nm. (b) Structure of Fe(CN)_6_^4–^(H_2_O)_160_ taken at the end of a simulated annealing molecular dynamics trajectory. Colors are overlaid according to the edges of the corresponding solvent shell divisions provided in (a).

In order to obtain an estimate for the radius of these clusters and the number of hydration shells, a molecular dynamics trajectory was performed for Fe(CN)_6_^4–^(H_2_O)_160_. A radial distribution function (Fig. 7a) shows three full solvation shells with the onset of a fourth shell. The first shell, centered ∼0.38 nm away from the ion, is the narrowest in width as a result of the strong interaction between water molecules and Fe(CN)_6_^4–^. The width of subsequent shells expands as the water molecules interact less strongly with the central ion and have more rotational and translational freedom in comparison to the rotationally constricted inner water molecules. Each of the shells are ∼0.25 nm away from one another, with the third shell at ∼0.87 nm the widest. The fourth shell is centered at ∼1.1 nm, where the first water molecules with the characteristic AAD orientation reside.

**Fig. 8:**
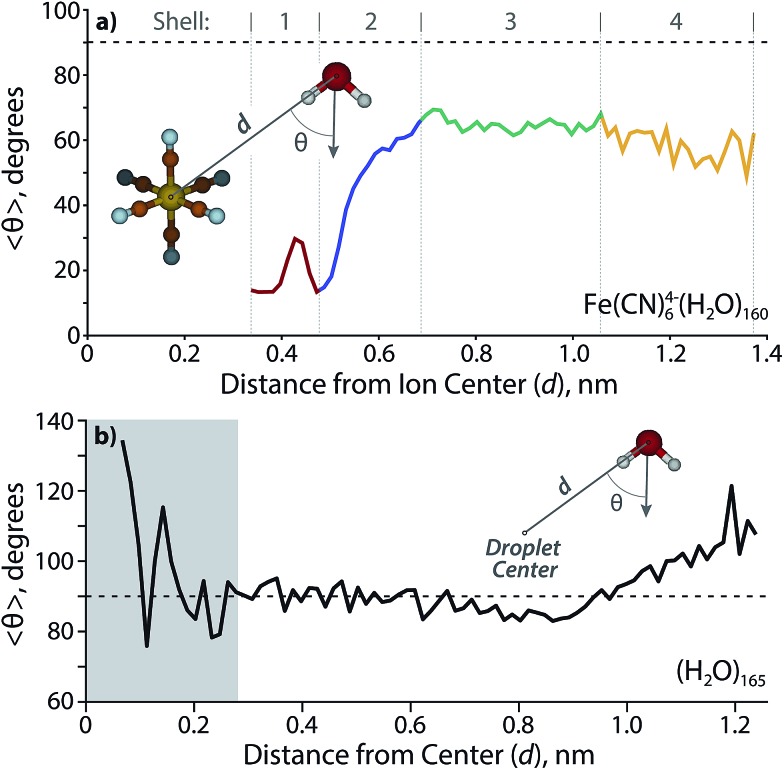
Average angle *θ* (depicted in insets) for water molecule orientation as a function of distance relative to (a) the solvated ion center in Fe(CN)_6_^4–^(H_2_O)_160_ and (b) the geometric center of (H_2_O)_165_. The horizontal dotted line corresponds to a water molecule orientation with the dipole perpendicular to the ion, θ = 90°. Water molecules are counted within a bin width for a radial thickness of 0.015 nm. The gray box in (b) extends to 0.28 nm, which represents the average O–O distance between water molecules and therefore is not indicative of any propagating trends.

A model of Fe(CN)_6_^4–^(H_2_O)_160_ at the end of the trajectory (Fig. 7b) provides a cross sectional view of the hydration shells with overlaid colors based on the radial distribution function. The distance from the center of the ion to the surface of the cluster for several structures along the trajectory results in a radius of 0.96 ± 0.05 nm, which is close to the distance obtained from a sphere using the density of bulk water (1.05 nm). The orientation of water molecules with respect to the solvated ion is shown in Fig. 8a. Water molecules within the first hydration shell interact strongly with the ion and have *θ* ≃ 20°. An increase in *θ* occurs until approximately 0.7 nm, which corresponds to the beginning of the third solvation shell. Although the average angle still faces inward toward the ion, water molecules in the second and third shells can hydrogen bond with a four-coordinate AADD motif, which enthalpically stabilizes a wider range of orientations than are possible within the first hydration shell. A value of *θ* ≃ 65° persists into the third shell until water molecules are 1.0–1.4 nm away from the ion. These are the outer shell surface water molecules, which are restricted in orientation due to diminished hydrogen-bonding partners. Although this is the distance where free O–H water molecules begin to emerge, the average water molecule is clearly orienting inward as far as 1.4 nm from the ion with a value of *θ* ≃ 55°, which is more acute with respect to the third shell as a result of surface constraints.

For comparison, data was also obtained for *θ* with respect to the center of a neutral nanodrop. The void space of Fe(CN)_6_^4–^ in Fe(CN)_6_^4–^(H_2_O)_160_ is ≃ 5 water molecules. Therefore, *n* = 165 was chosen in order to model a neutral nanodrop of comparable size (Fig. 8b). Water molecules are randomly oriented (*θ* ≃ 90°) up to ∼0.9 nm, where *θ* reaches a minimum of ∼85°. The water molecules between ∼1.0–1.24 nm are biased to face outward with respect to the center of the droplet, which is indicative of AAD water molecules that have a free O–H at the surface. The slight inward-facing bias between ∼0.7–0.95 nm is likely due to a restriction in orientation for water molecules in this shell in order to support the outward-facing motif of the surface water molecules. The furthest ion-oxygen distance reported for Fe(CN)_6_^4–^(H_2_O)_160_ (*d* = 1.37 nm) is slightly larger than that measured for (H_2_O)_165_ (*d* = 1.24 nm) as a result of Fe(CN)_6_^4–^ not being confined to the nanodroplet center.

## 4 Conclusions

Aqueous clusters of Fe(CN)_6_^4–^(H_2_O)_*n*_ with *n* ≥ 38 can be formed using nESI. Ferrocyanide is the highest charge density tetraanion that has been observed in the gas phase and measurements of aqueous nanodrops containing this anion provide a direct means for investigating how water stabilizes highly charged anions. Fe(CN)_6_^4–^(H_2_O)_38_ is the smallest cluster size observed directly by nESI and it dissociates exclusively by electron ejection under low-energy BIRD conditions. In addition to electron ejection, clusters of size *n* ≥ 39 can also dissociate by sequential loss of water molecules, which is the only dissociative pathway for clusters with *n* ≥ 41. Higher-energy dissociation conditions obtained through IRPD result in the entropically-favored loss of a water molecule to form Fe(CN)_6_^4–^(H_2_O)_37_, which is the smallest ferrocyanide cluster observed in these experiments.

The droplet radius for Fe(CN)_6_^4–^(H_2_O)_40_, the largest cluster unstable to electron ejection, is ∼0.66 nm. This corresponds to a nearly complete second hydration shell and is almost twice the radius observed for the trianion ferricyanide, which undergoes electron ejection with *n* ≤ 8 (*r* ≃ 0.38 nm).^21^ This is a clear illustration of ion–water interactions that extend beyond the inner coordination sphere. The necessity of solvation with *n* ≥ 41 is likely due to delocalization of the orbital density for valence electrons in Fe(CN)_6_^4–^(H_2_O)_*n*_, which must extend into a nearly complete second solvation shell. Stability obtained through ion–ion interactions results in a high propensity for ferrocyanide to form ion pairs with H^+^ and K^+^, which decrease the minimum solvation required for stability to electron ejection.

A free OH band in the IRPD spectrum of Fe(CN)_6_^4–^(H_2_O)_*n*_ appears between *n* = 142 and 162, indicating the ion–water interactions extend well beyond the second solvation shell. Remarkably, orientation of surface water molecules by the ion persists until *n* ≥ 162, which is significantly larger than that observed for the ferricyanide trianion (*n* ≥ 70). Based on structures from molecular modeling, clusters of size *n* = 160 have an average radius of ∼0.96 ± 0.05 nm, which extends into a fourth solvation shell. There is a linear trend between anionic charge state and cluster radii for the onset of AAD water molecules. Based on the single value for the Coulombic potential at the cluster surface obtained from these results, a small pentanionic species is predicted to orient water molecules up to clusters of size *n* ≃ 310, which have a radius of 1.3 nm.

The clusters in these experiments are equilibrated to cold temperatures (133 K) and the thermal motion of water molecules increases with temperature. At elevated temperatures, the entropic drive for surface water molecules to orient with free hydrogens will eventually predominate enthalpically favored hydrogen-bonding interactions. However, the perturbed water orientation observed up to nanometer length in these clusters is representative of interactions that, although diminished, will persist at warmer temperatures. These interactions are especially important in environments where water is scarce, such as in living cells and reverse micelles, and will manifest as changes to the dynamics of surrounding water molecules as well as freezing point depression.^40^ Long-distance forces originating from ions may also partially account for Hofmeister and other ion-related phenomena.
