# Supplementary material for: Nanometer patterning of water by tetraanionic ferrocyanide stabilized in aqueous nanodrops
Source: Chem Sci. 2016 Oct 17;8(2):1391–9. doi: 10.1039/c6sc03722d (PMC5361863; doi:10.1039/c6sc03722d)
Supplement: Supplementary file 1 [file SC-008-C6SC03722D-s001.pdf]

Electronic Supplementary Material (ESI) for Chemical Science.
This journal is © The Royal Society of Chemistry 2016

Supporting Information:

Nanometer Patterning of Water by Tetraanionic Ferrocyanide Stabilized in Aqueous Nanodrops

Matthew J. DiTucci and Evan R. Williams*

Department of Chemistry, University of California, Berkeley, California 94720–1460

E-mail: erw@berkeley.edu

Phone: +1 (510) 643-7161

*To whom correspondence should be addressed

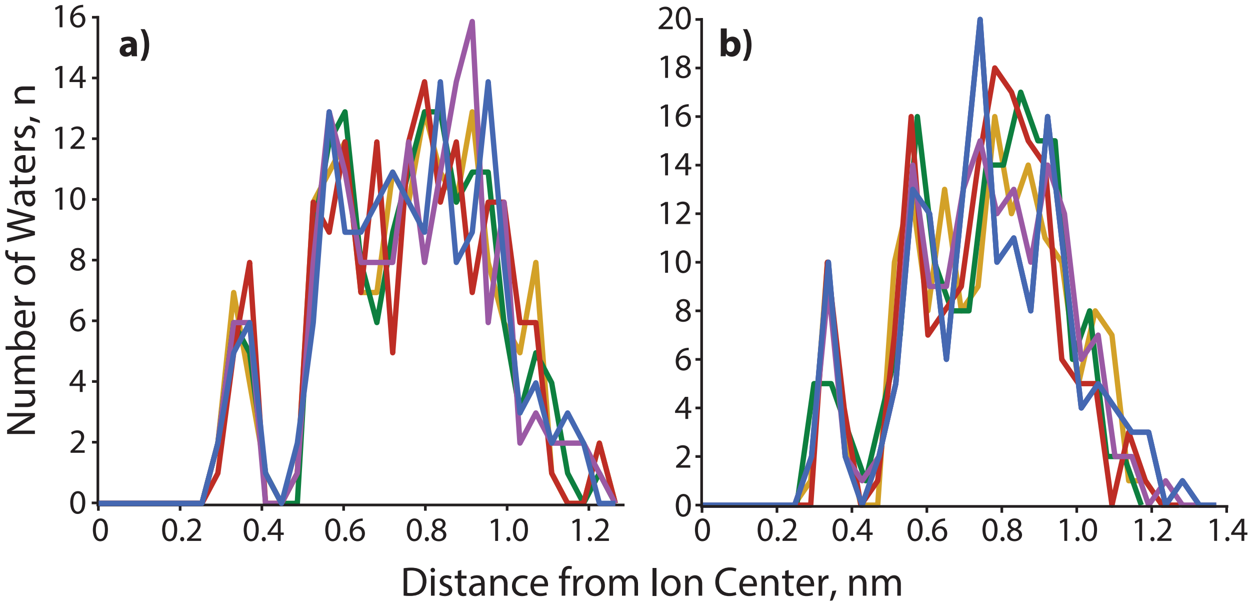

Figure 1: [Figure S1] Comparison of the radial distribution functions for five individual structures of $Fe(CN)_6^{4-}(H_2O)_{160}$, each plotted as a different color and taken from (a) 20 ps separations within the final 1 ns of a single annealing cycle and (b) the final structure within the last 1 ns of five separate annealing cycles. These plots illustrate that although the RMS displacement of a single water molecule at 133 K during the last 1 ns of a single cycle is $\sim 0.78 \pm 0.24$ Å, there is still an appreciable change to the overall nanodrop within this 20 ps timescale. The use of a simulated annealing cycle increases the RMS displacement for a single water molecule to $\sim 10.07 \pm 1.95$ Å, which indicates the potential energy of the system is sufficient to overcome local minima and exchange water molecules between hydration shells. The structure of a fourth solvation shell is apparent in the single distributions, which were used to calculate an average center of $\sim$1.10 nm (Figure 7).

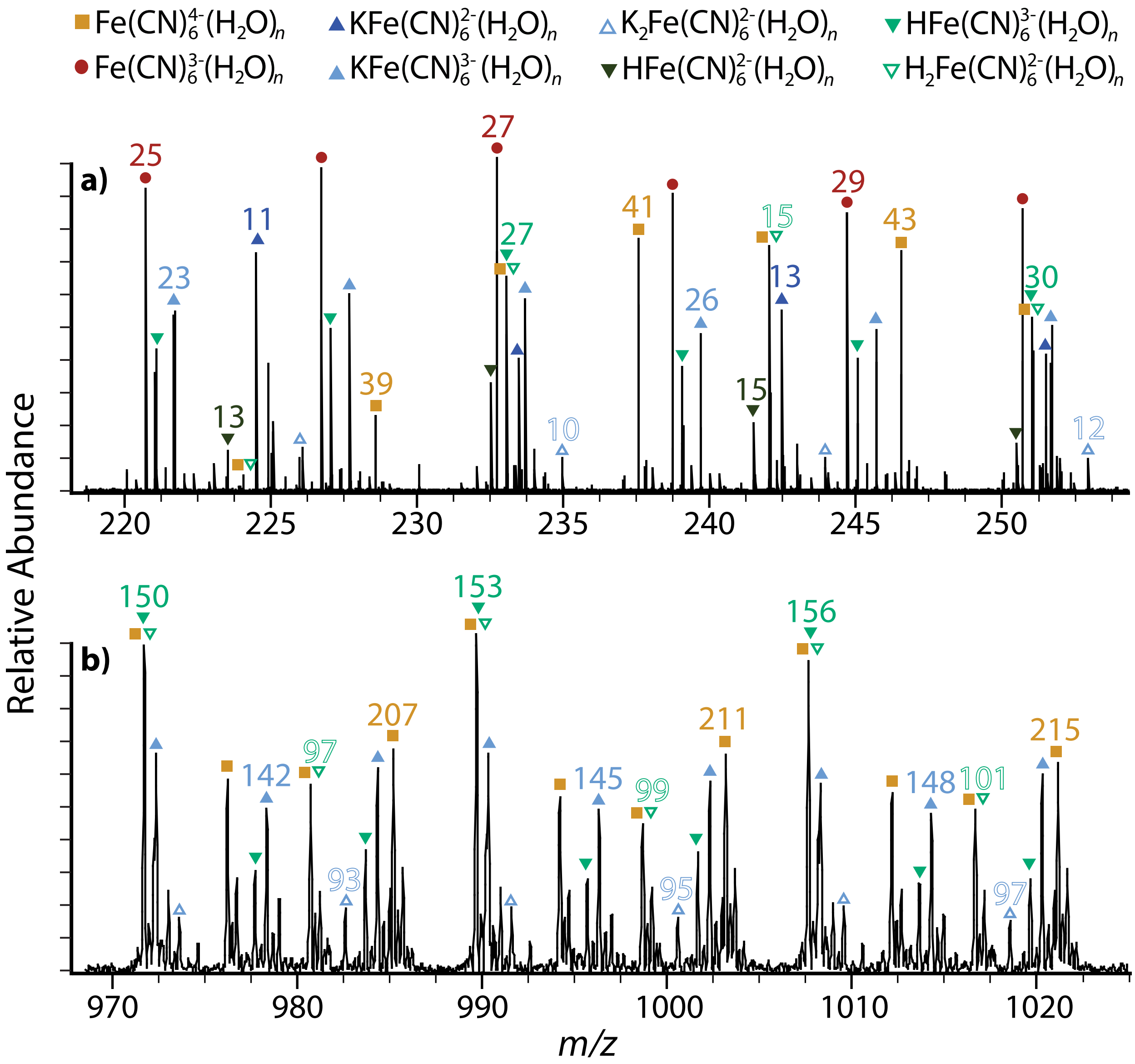

Figure 2: [Figure S2] Expanded regions from Figure 1 are shown to illustrate (a) the onset of ferrocyanide clusters and (b) the absence of trianion ferricyanide and associated adducts at large cluster sizes. Overlapping peaks are present for every second ferrocyanide cluster size with $H_2Fe(CN)_6^{2-}(H_2O)_n$ and every fourth ferrocyanide cluster size with $HFe(CN)_6^{3-}(H_2O)_n$.

Full Citation for Reference 6:

E. Harris, B. Sinha, D. Pinxteren, A. Tilgner, K. W. Fomba, J. Schneider, A. Roth, T. Gnauk, B. Fahlbusch, S. Mertes, T. Lee, J. Collett, S. Foley, S. Borrmann, P. Hoppe, H. Herrmann, *Science*, 2013, **340**, 727-730.

Full Citation for Reference 43:

P. J. Faustino, Y. Yang, J. J. Progar, C. R. Brownell, N. Sadrieh, J. C. May, E. Leutzinger, D. A. Place, E. P. Duffy, F. Houn, S. A. Loewke, V. J. Mecozzi, C. D. Ellison, M. A. Khan, A. S. Hussain, R. C. Lyon, *J. Pharm. Biomed. Anal.*, 2008, **47**, 114-125.

Full Citation for Reference 52:

Y. Shao, L. F. Molnar, Y. Jung, J. Kussmann, C. Ochsenfeld, S. T. Brown, A. T. B. Gilbert, L. V. Slipchenko, S. V. Levchenko, D. P. O'Neill, R. A. DiStasio, R. C. Lochan, T. Wang, G. J. O. Beran, N. A. Besley, J. M. Herbert, C. Y. Lin, T. Van Voorhis, S. H. Chien, A. Sodt, R. P. Steele, V. A. Rassolov, P. E. Maslen, P. P. Korambath, R. D. Adamson, B. Austin, J. Baker, E. F. C. Byrd, H. Dachsel, R. J. Doerksen, A. Dreuw, B. D. Dunietz, A. D. Dutoi, T. R. Furlani, S. R. Gwaltney, A. Heyden, S. Hirata, C. P. Hsu, G. Kedziora, R. Z. Khalliulin, P. Klunzinger, A. M. Lee, M. S. Lee, W. Liang, I. Lotan, B. Nair, B. Peters, E. I. Proynov, P. A. Pieniazek, Y. M. Rhee, J. Ritchie, E. Rosta, C. D. Sherrill, A. C. Simmonett, J. E. Subotnik, H. L. Woodcock, W. Zhang, A. T. Bell, A. K. Chakraborty, D. M. Chipman, F. J. Keil, A. Warshel, W. J. Hehre, H. F. Schaefer, J. Kong, A. I. Krylov, P. M. W. Gill, M. Head-Gordon, *Phys. Chem. Chem. Phys.*, 2006, **8**, 3172-3191.

Full Citation for Reference 54:

J. A. Fournier, C. T. Wolke, C. J. Johnson, M. A. Johnson, N. Heine, S. Gewinner, W. Schllkopf, T. K. Esser, M. R. Fagiani, H. Knorke, K. R. Asmis, *Proc. Natl. Acad. Sci. USA*, 2014, **111**, 18132-18137.

Cartesian Coordinates for Lowest Energy $Fe(CN)_6^{4-}(H_2O)_{160}$ Structure from Simulated Annealing Molecular Dynamics in Figure 7:

Fe: 0.00000 0.00000 0.00000
N: -0.00510 3.14710 0.00380
C: 0.01300 1.99980 0.01390
N: -3.15440 0.01050 -0.00130
C: -1.99620 -0.02470 0.01410
N: 0.00820 -0.00530 3.14030
C: -0.05410 -0.01190 1.98160
N: 0.00620 -3.15810 0.02200
C: 0.02040 -1.98050 -0.01120
N: 3.17960 0.00640 -0.00140
C: 1.99950 -0.02870 0.00300
N: 0.00440 0.00230 -3.15960
C: -0.00130 0.01500 -1.98770
O: 3.42240 -5.45580 5.01600
H: 4.20030 -5.49150 4.38860
H: 2.61210 -5.13670 4.52440
O: -2.24970 -1.59630 -2.13800
H: -1.38410 -1.21360 -2.46090
H: -2.50660 -1.15890 -1.27610
O: 0.39980 5.66850 6.62390
H: -0.39550 5.46150 7.19370
H: 0.62730 4.87000 6.06650
O: -1.80440 -10.03680 -2.61120
H: -2.71230 -9.62670 -2.52380
H: -1.57880 -10.14370 -3.57960
O: -2.43350 1.45330 10.23120
H: -2.41030 2.42340 10.47310
H: -1.49920 1.11640 10.11460
O: 1.26110 0.64930 -7.77070
H: 1.22010 1.28670 -7.00120
H: 0.56020 0.89110 -8.44160
O: 6.61150 2.71900 9.16870
H: 6.50930 2.05060 8.43200
H: 5.82460 2.66080 9.78310
O: 9.69260 -2.48910 -1.27970
H: 9.12090 -1.71420 -1.00990
H: 9.43630 -2.78820 -2.19880
O: 3.42930 0.37220 8.18000
H: 4.37470 0.38810 7.85450
H: 2.80690 0.41380 7.39850
O: 8.66270 2.79700 0.76450
H: 8.10860 1.99010 0.96920
H: 8.49130 3.08900 -0.17650
O: -8.64680 -0.08880 -1.52680
H: -8.85900 0.04630 -0.55890
H: -8.43100 -1.05160 -1.68950
O: -3.08480 -6.69080 0.97330
H: -3.33990 -5.79680 0.60470
H: -2.82540 -6.59350 1.93410
O: 8.00100 4.28760 7.30150
H: 7.57260 3.73160 8.01380
H: 7.30130 4.82930 6.83560
O: -5.34000 3.20140 4.30030
H: -5.81090 3.41170 5.15710
H: -5.19310 4.04550 3.78480
O: -0.72030 -8.90910 -5.19220
H: -0.99440 -8.44310 -6.03340
H: -0.72680 -8.25810 -4.43310
O: 8.58060 2.64640 5.28710
H: 8.60520 3.31130 6.03360
H: 7.96520 2.97320 4.56980
O: 0.12730 -7.62280 3.68180
H: 0.32950 -7.90940 2.74530
H: 0.47160 -6.69540 3.82820
O: 2.06600 -6.25440 -5.67490
H: 1.40470 -6.00650 -6.38280
H: 1.80930 -5.81910 -4.81190
O: -6.52750 -2.99480 -1.62230
H: -6.41270 -2.31210 -0.90060
H: -6.65590 -3.89690 -1.21040
O: 7.90050 -4.90410 -5.95660
H: 7.68160 -5.78760 -5.54250
H: 7.19350 -4.66340 -6.62170
O: 4.68400 1.79130 -3.48830
H: 3.98910 1.08440 -3.35660
H: 5.59200 1.39610 -3.34930
O: 10.06340 1.84880 2.99660
H: 9.54550 2.22260 2.22720
H: 9.57200 2.02980 3.84850
O: -1.08030 4.33560 -9.36280
H: -0.58070 4.59610 -8.53670
H: -2.02830 4.12310 -9.12590
O: 2.99010 -4.70860 1.20080
H: 3.34680 -5.07770 0.34260
H: 2.67230 -3.77190 1.05420
O: 4.26430 2.30710 -6.15620
H: 4.44920 2.21170 -5.17810
H: 4.64000 1.51950 -6.64470
O: 5.68310 -2.92690 -0.47330
H: 6.08250 -3.72960 -0.91620
H: 4.71590 -2.85820 -0.71760
O: 7.25870 -5.11290 -1.94410

H: 7.97900 -4.61180 -2.42370
H: 6.39020 -4.98910 -2.42410
O: 5.09790 2.93670 0.22840
H: 5.13880 2.40490 1.07430
H: 4.15370 2.96950 -0.09930
O: 0.49400 -1.80300 -7.20570
H: 0.76960 -0.87800 -7.46730
H: -0.48600 -1.81520 -7.00740
O: -6.97190 1.18760 3.26200
H: -6.46220 1.97710 3.60370
H: -7.06260 0.50870 3.99070
O: 0.25630 4.81400 -6.81410
H: -0.54600 5.09940 -6.28990
H: 0.65290 3.99600 -6.39750
O: -1.06060 -6.90290 -3.41610
H: -0.27270 -6.29070 -3.34980
H: -1.24030 -7.31290 -2.52180
O: 7.07930 3.47970 -1.55270
H: 6.40970 3.16130 -0.88170
H: 6.62330 4.05550 -2.23130
O: 1.68520 -1.68730 8.51720
H: 2.44740 -1.04320 8.58100
H: 1.85130 -2.32720 7.76690
O: -8.56910 -0.00910 1.33990
H: -8.09340 0.73170 1.81420
H: -8.77380 -0.74260 1.98810
O: 2.97690 3.96490 3.31640
H: 3.13030 4.63610 2.59120
H: 2.57500 3.13710 2.92490
O: 6.62770 4.99640 1.30620
H: 7.49420 4.82110 0.83880
H: 5.90660 4.44240 0.89020
O: 3.29530 5.27750 -1.52170
H: 2.54400 5.79460 -1.93180
H: 2.98960 4.34640 -1.32280
O: -3.45540 3.63460 -8.02870
H: -3.76580 3.80920 -7.09430
H: -3.39960 2.64800 -8.18250
O: -3.89530 -4.40300 -0.14570
H: -3.52360 -3.65200 0.40000
H: -3.78040 -4.19760 -1.11760
O: 2.31870 6.64530 -6.60790
H: 3.16570 6.14640 -6.42450
H: 1.64150 6.02060 -6.99670
O: 0.32810 0.76010 10.01250
H: 0.86350 -0.08410 9.98610
H: 0.59390 1.34740 9.24800
O: -6.25820 1.28230 -1.78170
H: -6.01110 1.39530 -0.81930
H: -7.19570 0.94030 -1.84720
O: -4.41830 3.41520 -2.60680
H: -3.54960 3.03340 -2.29130
H: -5.14480 2.73980 -2.48030
O: -4.62340 4.64630 -0.09370
H: -4.71680 4.44620 -1.06900
H: -4.26710 3.84040 0.37930
O: -0.99410 -1.43740 7.64520
H: -0.10860 -1.41150 8.10900
H: -0.96480 -2.11270 6.90820
O: -1.19880 4.98020 -2.36940
H: -1.57060 4.05730 -2.46880
H: -1.43220 5.33800 -1.46520
O: 0.67740 3.68850 4.83290
H: 1.51370 3.92690 4.33910
H: 0.01770 3.28780 4.19710
O: 4.42670 -2.09080 -4.07470
H: 3.87360 -1.36900 -3.65890
H: 5.38640 -1.81000 -4.08710
O: 2.08200 -2.00490 1.72710
H: 1.19590 -2.27430 1.34990
H: 2.39180 -1.16430 1.28270
O: 2.94490 5.81970 1.14320
H: 1.98190 6.02350 0.96680
H: 3.40780 5.62340 0.27890
O: 0.87810 -7.78300 0.95370
H: 1.63440 -7.13080 0.90260
H: 0.25720 -7.63450 0.18400
O: 7.27880 1.09580 -2.83960
H: 7.35070 1.97850 -2.37510
H: 7.32420 0.35930 -2.16470
O: 6.40620 3.44190 3.70590
H: 6.58710 3.94200 2.85900
H: 5.87900 2.61750 3.50000
O: 0.47480 6.89020 0.31370
H: -0.48190 6.60200 0.27080
H: 0.84870 6.94340 -0.61230
O: 1.88030 0.63480 5.91710
H: 2.19120 -0.10770 5.32380
H: 0.88330 0.69980 5.87490
O: 1.00080 6.54180 -2.40190
H: 0.93610 7.16780 -3.17910
H: 0.22830 5.90710 -2.42010
O: 4.80380 -8.50830 -3.65060
H: 3.91240 -8.85220 -3.94570
H: 4.87430 -8.57570 -2.65540
O: 4.75970 -6.38730 -5.46490
H: 4.94390 -7.14950 -4.84440
H: 3.77440 -6.31270 -5.61800
O: -3.10760 2.46560 1.09360
H: -2.74330 1.73420 0.51700

H: -2.35540 2.91900 1.57170
O: 7.40640 0.68820 1.69290
H: 6.62860 1.02230 2.22510
H: 8.02230 0.17370 2.28970
O: -4.86290 5.51600 2.73750
H: -3.89020 5.29940 2.81980
H: -5.13510 5.46710 1.77650
O: -2.16330 5.87550 0.17540
H: -2.10100 5.74210 1.16450
H: -3.12480 5.89330 -0.09870
O: -1.61620 5.79300 -5.08530
H: -1.60540 5.60050 -4.10410
H: -2.50910 5.54800 -5.46320
O: -2.15440 5.14580 2.97330
H: -1.72720 5.76400 3.63300
H: -1.57770 4.33780 2.85180
O: 2.70360 -1.36570 4.36620
H: 2.20100 -1.57220 3.52680
H: 3.68100 -1.30620 4.16340
O: -3.55130 -3.81140 -2.92990
H: -4.36030 -3.39470 -3.34470
H: -2.87780 -3.10090 -2.72620
O: -5.58760 -2.40920 -3.96710
H: -5.13090 -1.52740 -3.84930
H: -6.09580 -2.63490 -3.13600
O: 1.18280 2.56700 -3.00990
H: 1.73450 2.76520 -2.19980
H: 0.65060 1.73470 -2.85540
O: 4.03390 -0.17710 -7.61810
H: 3.71500 -1.00580 -7.15820
H: 3.28360 0.22070 -8.14620
O: 4.68470 -8.52650 -0.81220
H: 5.24830 -8.23930 -0.03770
H: 4.00280 -9.18840 -0.50090
O: 7.82940 -0.04370 5.22020
H: 8.49390 -0.53080 4.65350
H: 8.15130 0.88880 5.38430
O: 4.87820 1.44340 2.72570
H: 3.91290 1.50880 2.47290
H: 5.03060 0.60250 3.24500
O: -0.54130 -2.87070 3.37710
H: -1.36610 -2.98260 2.82290
H: -0.11950 -1.98740 3.17230
O: -5.59830 6.16590 5.40810
H: -5.35260 6.10860 4.44050
H: -4.83830 6.56390 5.92200
O: -0.08780 -4.74560 -6.82390
H: -0.47680 -4.43110 -5.95810
H: 0.35190 -3.97790 -7.29020
O: -3.75610 -1.67810 7.32140
H: -2.79290 -1.45550 7.47210
H: -4.01950 -1.40880 6.39510
O: -2.98980 7.04000 6.55780
H: -2.59470 6.49010 7.29370
H: -2.30420 7.18350 5.84410
O: 0.51230 7.57480 -4.77900
H: 1.27170 7.35230 -5.39040
H: -0.25930 6.96870 -4.97160
O: 9.32940 -3.29560 1.36000
H: 9.17420 -4.27010 1.19790
H: 9.68480 -2.87160 0.52690
O: -3.79770 2.86260 7.56200
H: -4.00210 1.99490 8.01500
H: -3.46070 2.68480 6.63750
O: 8.58340 -3.45820 -3.75860
H: 8.04740 -2.62780 -3.91100
H: 8.58920 -4.00900 -4.59320
O: 2.20170 1.55930 2.22710
H: 1.38520 1.18120 2.66350
H: 2.38220 1.06790 1.37510
O: -4.27180 -0.04460 -3.29380
H: -4.91910 0.44960 -2.71350
H: -3.69310 -0.62910 -2.72510
O: -1.16930 -7.93190 -0.92470
H: -1.95180 -7.54530 -0.43660
H: -1.38060 -8.86830 -1.20480
O: 1.21730 2.79740 11.83930
H: 1.02320 1.83620 11.64310
H: 2.10650 3.04330 11.45360
O: -1.93870 5.01740 8.08660
H: -1.78520 4.70590 9.02440
H: -2.51840 4.35860 7.60720
O: -2.90580 0.36980 3.05460
H: -3.17370 1.06650 2.38920
H: -1.98770 0.03770 2.83820
O: 2.54120 -3.14750 6.39170
H: 2.52390 -2.44680 5.67840
H: 3.22630 -3.83910 6.16290
O: -0.94320 3.83700 10.36090
H: -0.35710 3.92260 11.16660
H: -0.44540 3.36160 9.63560
O: 1.08210 -10.13700 -2.30410
H: 0.09640 -10.22340 -2.44810
H: 1.49020 -9.64910 -3.07570
O: 3.07710 7.27390 3.40090
H: 3.02020 6.80250 2.52080
H: 4.02800 7.52310 3.58470
O: 3.12560 -2.63120 -6.61290
H: 3.34230 -2.63150 -5.63660
H: 2.19460 -2.29200 -6.74760

O: -2.44620 -2.42950 1.32320
H: -2.68770 -1.50960 1.01430
H: -1.58740 -2.71030 0.89470
O: -5.45850 -7.85900 0.06630
H: -4.58940 -7.65010 0.51470
H: -6.03950 -7.04520 0.07000
O: -7.07630 -0.68210 5.40000
H:: -7.10440 -0.12310 6.22870
H: -6.12990 -0.93720 5.20190
O: -4.13510 -8.85670 -2.25360
H: -4.17700 -7.95240 -2.67860
H: -4.65740 -8.84870 -1.40090
O: -4.50390 0.53130 8.86170
H: -4.45530 -0.39480 8.48760
H: -3.64620 0.74800 9.32780
O: 2.61360 2.65010 -0.72320
H: 2.80040 1.67590 -0.85000
H: 1.70120 2.76620 -0.33080
O: -8.69900 -1.73610 3.36030
H: -8.07220 -2.43950 3.02490
H: -8.38690 -1.40950 4.25240
O: -3.11550 1.88050 5.20810
H: -2.90820 1.25190 4.45850
H: -3.92640 2.41830 4.97740
O: -0.68010 2.85500 2.56590
H: -0.22880 2.97430 1.68160
H: -0.75080 1.87970 2.77520
O: -6.48150 -5.12080 0.22600
H: -5.49820 -4.94360 0.26870
H: -6.94460 -4.60580 0.94730
O: 3.28600 3.86990 7.58760
H: 3.60940 3.18870 6.93080
H: 3.19390 4.75510 7.13160
O: -6.86290 1.11480 7.33270
H: -6.82520 2.11260 7.27700
H: -6.21570 0.79490 8.02470
O: -6.28720 3.94790 6.88180
H: -5.39670 3.92410 7.33630
H: -6.40040 4.82540 6.41590
O: -2.41980 -5.78200 3.87410
H: -2.53750 -5.40360 4.79220
H: -1.77480 -6.54540 3.90840
O: 5.89700 7.17510 2.88500
H: 6.17520 6.88240 3.79980
H: 6.13580 6.46630 2.22120
O: 6.55780 -3.03800 2.15740
H: 6.21900 -2.78010 1.25260
H: 7.52280 -3.29210 2.09210
O: -2.89170 0.67640 -8.20390
H: -3.10020 -0.22040 -8.59400
H: -2.71730 0.58110 -7.22380
O: 4.76580 -4.66580 -3.12910
H: 4.62880 -3.72540 -3.44050
H: 4.78050 -5.27940 -3.91860
O: 2.45370 -9.85670 0.08500
H: 2.04840 -10.11690 -0.79140
H: 1.79190 -9.32510 0.61360
O: 9.33150 -1.07450 3.10070
H: 10.10420 -0.53640 2.76410
H: 9.39030 -2.00480 2.73860
O: 1.23780 -4.94290 3.43730
H: 0.63780 -4.14380 3.39680
H: 1.79800 -4.98140 2.60980
O: 5.09770 4.47520 -3.25480
H: 4.90250 3.49700 -3.32560
H: 4.47320 4.89290 -2.59490
O: -6.70270 -3.23790 2.51230
H: -5.86130 -3.63860 2.87490
H: -6.47550 -2.57310 1.80070
O: -3.76910 -6.46950 -3.46750
H: -2.79190 -6.66710 -3.54520
H: -3.89510 -5.52260 -3.17160
O: 2.91770 -8.32400 4.42840
H: 1.91860 -8.32030 4.38750
H: 3.23260 -7.53470 4.95550
O: 0.44990 8.10560 2.83020
H: 1.36980 8.04850 3.21820
H: 0.48250 7.86930 1.85900
O: -1.10080 -3.52760 -4.48020
H: -0.43210 -3.21880 -3.80380
H: -1.87980 -3.94510 -4.01230
O: 5.97290 -7.59750 1.50470
H: 5.86650 -6.81150 2.11360
H: 5.57880 -8.40910 1.93600
O: 3.66230 2.67530 10.00120
H: 3.52380 3.33100 9.25900
H: 3.68090 1.74790 9.62750
O: 5.03350 -5.20150 2.85850
H: 4.28080 -5.14480 2.20260
H: 5.60020 -4.38070 2.78740
O: 3.16270 -6.24880 -1.44170
H: 3.68820 -7.07210 -1.22720
H: 3.77800 -5.54220 -1.79120
O: 4.51110 4.97910 -5.88980
H: 4.91360 5.02120 -4.97530
H: 4.48650 4.02870 -6.19970
O: 2.01970 -8.87420 -4.49540
H: 2.27770 -7.95480 -4.79220
H: 1.17250 -9.14630 -4.95170
O: -2.21110 -1.88310 -6.42970

H: -1.82060 -2.50470 -5.75060
H: -2.36350 -0.98700 -6.01270
O: 8.18250 -5.90770 0.66790
H: 7.95060 -5.60890 -0.25780
H: 7.64920 -6.72150 0.89890
O: -2.63250 0.50560 -5.39950
H: -2.00970 1.25810 -5.18550
H: -3.29080 0.39580 -4.65470
O: 0.58310 -2.61420 -2.66320
H: 0.64470 -1.64660 -2.90800
H: 0.29060 -2.69900 -1.71070
O: -3.71580 3.53030 -5.33210
H: -2.77930 3.19440 -5.23080
H: -4.14400 3.60010 -4.43110
O: 0.68740 2.80330 8.23110
H: 1.55370 3.06430 7.80530
H: 0.10000 2.36540 7.55050
O: -0.72640 1.53910 -9.72210
H: -1.64120 1.32120 -9.38210
H: -0.57220 2.52490 -9.65570
O: -1.88150 2.31120 -1.93430
H: -1.19700 2.51630 -1.23470
H: -1.87740 1.33000 -2.12710
O: -4.47920 -1.33100 4.62390
H: -4.44050 -2.19220 4.11710
H: -4.05650 -0.60540 4.08100
O: -0.20550 -3.36350 5.93340
H: 0.76440 -3.48210 6.14630
H: -0.31940 -3.25720 4.94560
O: 6.61690 -1.45160 -7.22280
H: 5.77130 -0.96930 -7.45130
H: 6.44390 -2.43630 -7.20210
O: 7.34720 -0.72510 -0.75030
H: 6.69290 -1.47740 -0.67330
H: 7.34150 -0.19050 0.09480
O: -3.84400 -6.39810 -6.24910
H: -4.00910 -5.44080 -6.48650
H: -3.90560 -6.51080 -5.25740
O: 5.89250 1.01100 7.13720
H: 5.43060 1.56620 6.44560
H: 6.64040 0.50200 6.71110
O: 1.50740 2.49370 -5.69630
H: 2.39950 2.54750 -6.14490
H: 1.62850 2.55310 -4.70540
O: -6.21470 -1.30850 0.58230
H: -5.41900 -0.70280 0.58120
H: -7.03630 -0.77900 0.79310
O: 2.93960 -2.69370 -1.22770
H: 2.28810 -2.91590 -1.95300
H: 2.58870 -1.92900 -0.68720
O: 2.79710 -0.16920 -2.80750
H: 2.89090 -0.30140 -1.82070
H: 1.83250 -0.03770 -3.03600
O: 3.04580 6.17110 6.02300
H: 2.98330 6.45810 5.06710
H: 2.13780 6.20290 6.44070
O: 4.00570 2.30960 5.43170
H: 3.16710 1.77080 5.51180
H: 3.90880 2.96800 4.68530
O: -2.82620 -4.46120 6.17640
H: -1.85660 -4.22730 6.24890
H: -3.37810 -3.71130 6.54100
O: 5.87540 5.77280 5.44530
H: 4.91770 5.92620 5.68880
H: 5.94260 4.97970 4.84000
O: -0.79740 1.03200 6.52110
H: -1.52200 1.38850 5.93140
H: -1.05630 0.12640 6.85700
O: -4.29530 -3.80670 -6.16710
H: -4.81320 -3.33510 -5.45340
H: -3.64570 -3.17040 -6.58320
O: 1.04780 -5.33500 -3.00060
H: 1.72580 -5.69880 -2.36190
H: 0.85580 -4.38000 -2.77470
O: -1.04400 7.02430 4.70690
H: -0.51300 7.52920 4.02650
H: -0.42700 6.64320 5.39550
O: 4.33260 -9.53240 2.46400
H: 3.82160 -9.06070 3.18260
H: 3.71040 -10.11330 1.93910
O: 5.34580 -4.29050 -7.11720
H: 4.51970 -3.72870 -7.16130
H: 5.15700 -5.12030 -6.59210
O: 7.18180 -1.18680 -4.64010
H: 7.06030 -1.14160 -5.63160
H: 7.51100 -0.30440 -4.30400
O: 5.52880 -0.95300 3.85090
H: 5.81910 -1.73240 3.29560
H: 6.31610 -0.58420 4.34510
O: -1.11910 2.64300 -4.55950
H: -0.16860 2.59340 -4.86630
H: -1.15410 2.54170 -3.56520
O: -4.13630 -3.70160 3.01280
H: -3.61490 -4.52110 3.25060
H: -3.68840 -3.23030 2.25300
O: -1.39760 -7.13030 -7.15770
H: -0.85580 -6.29020 -7.13140
H: -2.34700 -6.92210 -6.92260
O: -5.59160 1.51770 0.79430
H: -4.67660 1.90460 0.90870
H: -5.99990 1.35650 1.69280
